# Early initiation of venovenous ECMO for drowning-associated refractory hypoxemia after cardiac arrest: a case report

**DOI:** 10.1186/s12245-026-01251-9

**Published:** 2026-05-09

**Authors:** Soichiro Kano, Yoshinori Kakino, Hirotaka Asano, Yugo Wakayama, Erika Takada, Hajime Ichiryu, Genki Naruse, Yosuke Mizuno, Tetsuya Fukuta, Takuya Matsumoto, Takahito Miyake, Shozo Yoshida, Hideshi Okada

**Affiliations:** 1 Advanced Critical Care Center, Gifu University Hospital, Gifu, Japan; 2 Nakatsugawa Municipal General Hospital, Nakatsugawa, Japan; 3 Abuse Prevention Emergency Medicine, Gifu University Graduate School of Medicine, Gifu, Japan; 4 Department of Emergency and Disaster Medicine, Gifu University Graduate School of Medicine, 1-1 Yanagido, Gifu, 501-1194 Japan; 5 Center for One Medicine Innovative Translational Research (COMIT), Gifu University, Gifu, Japan

**Keywords:** Drowning, Venovenous extracorporeal membrane oxygenation, Case report

## Abstract

**Background:**

Drowning-associated cardiac arrest is associated with high mortality and poor neurological outcomes. The appropriate prognosis prediction and determination of a treatment plan are crucial.

**Case presentation:**

A 19-year-old Japanese male was playing in a river. His friend found him drowning and immediately pulled him out. He experienced cardiac arrest. His friend called for emergency services and performed bystander cardiopulmonary resuscitation. When the emergency service later arrived at the scene, the patient was in a state of return of spontaneous circulation. The patient was transported to our hospital by air ambulance. He was subsequently admitted to the intensive care unit and later discharged without residual higher brain dysfunction as a result of multidisciplinary treatment, including venovenous extracorporeal membrane oxygenation (V–V ECMO).

**Conclusions:**

In drowning-associated cardiac arrest with rapidly progressive hypoxemia, early initiation of V–V ECMO as part of comprehensive post–cardiac arrest management may contribute to favorable neurological outcomes.

## Background

In Japan, 8,993 deaths due to “accidental drowning and submersion” were reported in 2023. It consistently ranks high as a cause of death among individuals aged 5–19 years and is increasing among older individuals during bathing [1]. Drowning-associated cardiac arrest is associated with substantial mortality and neurological morbidity despite advances in resuscitation and critical care [2]. Survivors of drowning may experience persistent neurological impairment depending on the duration of hypoxia and the quality of resuscitation [2].

Under these circumstances, predicting biological and neurological prognoses and determining an appropriate treatment strategy at an early stage are crucial. We successfully treated a patient with severe acute respiratory distress syndrome (ARDS) that developed after resuscitation from cardiac arrest due to river drowning, without complications such as higher brain dysfunction, through early administration of venovenous extracorporeal membrane oxygenation (V–V ECMO) and multidisciplinary treatment.

## Case presentation

The patient, a previously healthy 19-year-old male, was playing in a river with a friend. At 4:02 PM, his friend who was nearby found him drowning and pulled him out. The drowning time was estimated to be less than 5 min; however, the patient was in cardiac arrest. The friend called for emergency medical assistance, and bystander cardiac resuscitation was performed with verbal instructions by paramedics over the phone. At 4:27 PM, when the emergency medical service arrived and assessed the patient, he regained spontaneous circulation.

The patient was intubated, and a bone marrow needle was placed by a physician in an emergency vehicle from the nearest hospital. After requesting transportation to our hospital, our air physician evaluated the patient at 5:22 PM. At that time, the Glasgow Coma Scale (GCS) score was 6 (E1VTM4), and pupils were 4 mm bilaterally, and pupillary light reflexes were present and prompt. The patient was deemed to require intensive care and was transported to our hospital by ambulance.

The patient arrived at the emergency room at 6:02 PM. On arrival, the patient had a GCS score of 6 (E1VT M4), pupils 4 mm bilaterally with preserved pupillary light reflexes, the core body temperature of 35.4 °C, a heart rate of 136 beats/min, and a blood pressure of 142/75 mmHg. Despite endotracheal intubation and assisted ventilation, SpO₂ was not detectable. At 6:09 PM, an arterial blood gas test revealed severe hypoxemia and lactic acidosis, with a PaO2 of 38.1 mmHg, a pH of 7.080, and a lactate level of 77 mg/dL (Table 1). Blood tests revealed elevated levels of cardiac enzymes, liver enzymes, and lactate dehydrogenase, along with hyponatremia (Table 1).

**Table 1:** Laboratory findings on admission

Complete blood count			Biochemical examination		
White blood cell	8260	/µL	Total protein	6.2	g/dL
Red blood cell	578	×10^6^/µL	Albumin	3.4	g/dL
Hemoglobin	16.4	g/dL	Creatine kinase	540	IU/L
Hematocrit	48.5	%	Creatine kinase MB	9	IU/L
Platelet	33.2	×10^4^/µL	Troponin T	0.066	mg/dL
			Aspartate transaminase	204	IU/L
**Coagulation status**			Alanine transaminase	126	IU/L
Activated partial thromboplastin time	31.8	sec	Lactate dehydrogenase	1220	IU/L
Prothrombin time (PT)	13.2	%	Alkaline phosphatase	108	IU/L
PT-international normalized ratio	1.23		Cholinesterase	277	IU/L
Fibrinogen	176	mg/dL	Amylase	727	IU/L
			Creatinine	1.16	mg/dL
**Arterial blood gas**			Urea nitrogen	17.3	mg/dL
F_I_O_2_			Total bilirubin	1	mg/dL
pH	7.08		Sodium	129	mEq/L
PaCO_2_	57.8	mmHg	Potassium	3.8	mEq/L
PaO_2_	38.1	mmHg	Chloride	95	mEq/L
HCO_3_^−^	17.1	mmol/L	Calcium	7.2	mEq/L
Base excess	-13.7		Blood glucose	239	mg/dL
Lactate	77	mg/dL	C-reactive protein	0.17	mg/dL

**Fig. 1:**
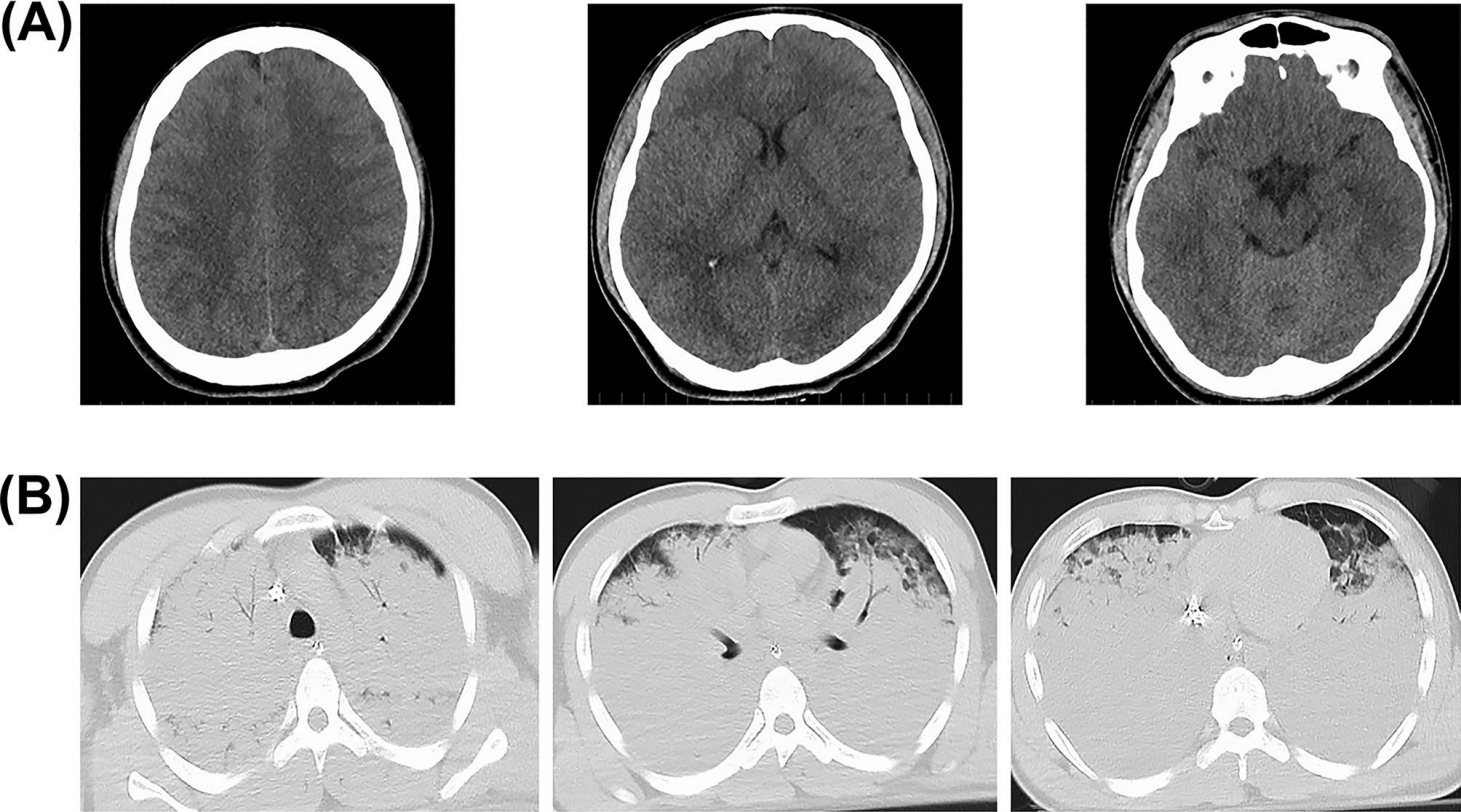
Computed tomography of (**a**) head and (**b**) chest on admission. **a**. Corticomedullary junction of the brain is clearly defined. **b**. Pulmonary edema or atelectasis in almost all areas of the lungs

Mechanical ventilation was initiated immediately with a FiO₂ of 1.0, a PEEP of 12 cmH₂O, and a tidal volume of 400 mL (approximately 6 mL/kg). Despite these settings, profound hypoxemia persisted. In addition to severe hypoxemia, the patient demonstrated marked metabolic acidosis (pH 7.080) and elevated lactate levels, indicating significant systemic hypoperfusion in the context of post–cardiac arrest syndrome. Given the combined respiratory and metabolic instability, the decision to initiate V–V ECMO was made in the emergency room. At our institution, V–V ECMO is considered in cases of refractory severe hypoxemia (PaO₂/FiO₂ < 80 despite optimal mechanical ventilation) or progressive metabolic deterioration in the context of post–cardiac arrest syndrome. ECMO support was started at 6:47 PM, approximately 45 min after arrival and prior to chest CT confirmation of ARDS. V–V ECMO was established using a CAPIOX^®^ SP-200 centrifugal pump system (Terumo, Tokyo, Japan), with an initial extracorporeal blood flow of 4.0 L/min and sweep gas FiO₂ of 0.9. After initiation of V–V ECMO, the patient’s PaO₂ improved to 97 mmHg, pH to 7.264, and lactate level decreased to 53 mg/dL at 7:00 PM. During ECMO support, ultraprotective ventilation was applied using the PC-BiPAP mode with a FiO₂ of 0.5, a PEEP of 12 cmH₂O, an inspiratory pressure of 22 cmH₂O, and a respiratory rate of 12 breaths/min, resulting in tidal volumes of approximately 60 mL. Prone positioning was not performed during ECMO therapy in accordance with institutional practice. Whole-body computed tomography (CT) was performed at 11:50 PM. The head had clear corticomedullary borders in the brain, and chest CT revealed pulmonary edema or atelectasis in almost all lung areas (Fig. 1). Chest CT on admission demonstrated diffuse bilateral infiltrates consistent with acute lung injury secondary to freshwater aspiration. Empirical antibiotic therapy with piperacillin/tazobactam (4.5 g every 8 h) was initiated due to the risk of aspiration-associated pneumonia. Initial sputum cultures revealed no significant bacterial growth. On hospital day 7, antibiotics were de-escalated to ampicillin/sulbactam (3.0 g every 6 h). However, the sputum culture obtained on hospital day 9 subsequently revealed *Aeromonas hydrophila*, and the antibiotic therapy was changed to ceftriaxone (2.0 g every 24 h). Antibiotics were discontinued on hospital day 20 following clinical improvement.

**Fig. 2:**
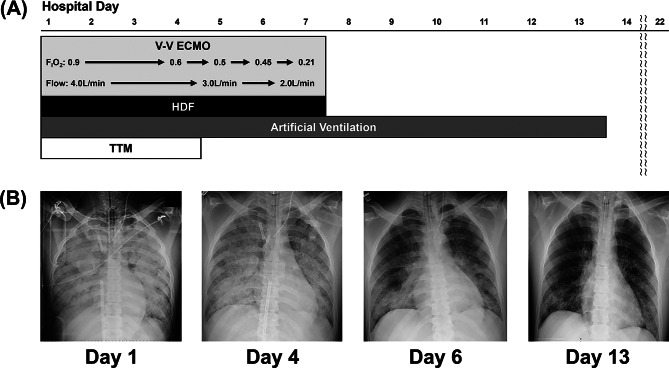
Clinical course. **a**. Schema of clinical course. **b**. Chest radiography on days 1 and 4 (end of TTM), 6 (end of V–V ECMO), and 13 (end of mechanical ventilation). V–V ECMO, venovenous extracorporeal membrane oxygenation; F_I_O_2_, fraction of inspiratory oxygen; HDF, hemodiafiltration; TTM, targeted temperature management

The patient was admitted to the intensive care unit for multidisciplinary management, including V–V ECMO support, blood purification therapy for systemic inflammatory modulation, and targeted temperature management for brain protection. Blood purification therapy was performed concurrently with ECMO. On hospital days 1–3, intermittent hemodiafiltration (HDF) was conducted for 8 h during the daytime, followed by sustained low-efficiency dialysis (SLED) for 16 h overnight. On days 4–6, HDF was performed for 8 h daily. A high-flux dialyzer (FDZ21) was used. Targeted temperature management was initiated with a target temperature of 34 °C and maintained for 24 h. Core body temperature was continuously monitored via a bladder thermistor. Temperature control was achieved using the heat exchanger integrated into the ECMO circuit without additional external cooling devices. Controlled rewarming to 37 °C was performed gradually over 48 h (Fig. 2a). Oxygenation gradually improved (Fig. 2b), and the patient was successfully weaned from V–V ECMO on day 7. The patient was extubated and withdrawn from artificial ventilation on day 13. Brain magnetic resonance imaging on day 14 revealed no signs of hypoxic encephalopathy. Oxygen therapy was completed on day 16.

At ICU discharge, the patient was fully conscious with a Glasgow Coma Scale score of 15 and a Modified Rankin Scale (mRS) score of 3. Before hospital discharge, a detailed cognitive assessment performed in the general ward revealed an Addenbrooke’s Cognitive Examination–Revised (ACE-R) score of 93/100 and a Frontal Assessment Battery (FAB) score of 16/18, indicating preserved higher brain function with only minimal impairment. On day 22, the patient was discharged home with a mRS of 1 and categorized as Cerebral Performance Category 1 (good cerebral performance). During outpatient follow-up at 3 and 6 months, the patient had a mRS of 0 and no neurological deficits.

## Discussion and conclusions

Drowning-induced lung injury results from the aspiration of water, leading to surfactant disruption, increased alveolar–capillary permeability, and noncardiogenic pulmonary edema. These changes cause severe ventilation–perfusion mismatch and intrapulmonary shunting, which may rapidly progress to acute respiratory distress syndrome (ARDS) [2]. In such cases, profound shunt physiology may become refractory to conventional mechanical ventilation, providing a physiological rationale for V–V ECMO support.

The patient experienced cardiac arrest but was found quickly, and the drowning time was estimated to have been within 5 min. The International Liaison Committee on Resuscitation compared the factors affecting neurological outcomes and survival in drowning patients in terms of immersion time (short, 5–6 min; intermediate, 10 min; and long, 15–25 min) and reported that shorter immersion times were associated with better outcomes [3].

In the present case, the PaO₂/FiO₂ ratio of 38.1 fulfilled the conventional ARDS criteria for V–V ECMO, which is consistent with the thresholds reported in the EOLIA trial [4]. However, patients with cardiac arrest were excluded from that study. Drowning complicated by cardiac arrest represents a more complex pathophysiological condition characterized not only by severe hypoxemia but also by systemic ischemia–reperfusion injury and metabolic acidosis as part of post–cardiac arrest syndrome (PCAS) [3]. These additional factors may accelerate clinical deterioration and increase the risk of secondary organ injury, particularly to the brain. In this context, early initiation of V–V ECMO may be considered not only on the basis of oxygenation parameters but also on the broader metabolic and neurological risk profile. Recent registry data suggest that extracorporeal life support may improve survival in selected patients with drowning-associated cardiac arrest [5]. In the present case, V–V ECMO was initiated in the emergency room prior to CT confirmation of ARDS, reflecting a clinical strategy prioritizing rapid physiological stabilization over formal diagnostic confirmation. In drowning-associated cardiac arrest, systemic inflammation may be triggered not only by aspiration-induced lung injury but also by ischemia–reperfusion injury as part of post–cardiac arrest syndrome. In this case, blood purification therapy was applied with the aim of modulating systemic inflammation, in addition to its role in supporting organ function. However, its direct effect on lung injury remains uncertain. Lung-protective ventilation and V–V ECMO support were considered the primary strategies for managing respiratory failure. In freshwater drowning, aspiration of hypotonic water may lead to electrolyte disturbances and is associated with an increased risk of infections by specific organisms such as Aeromonas hydrophila. In the present case, mild hyponatremia and subsequent Aeromonas infection were observed, consistent with freshwater exposure. In addition, environmental and regional factors, including water quality and local healthcare systems, may influence both clinical presentation and management strategies in drowning-associated cardiac arrest.

The favorable neurological outcome in this case was likely multifactorial. The short estimated immersion time, prompt resuscitation at the scene, and relatively preserved core body temperature on arrival, as well as rapid initiation of comprehensive post–cardiac arrest management—including targeted temperature management, kidney replacement therapy, and V–V ECMO—may all have contributed to recovery. Importantly, early clinical decision-making in the emergency setting allowed timely escalation of support in a rapidly deteriorating patient. Environmental factors and early comprehensive management, including rapid decision-making and timely initiation of advanced supportive therapies, were also considered important in determining the treatment strategy.

Overall, when a favorable neurological prognosis is anticipated—such as in patients with short immersion times—aggressive multidisciplinary management, including timely initiation of V–V ECMO, may contribute to improved survival and neurological recovery.

### Limitations

This report describes a single case from a single center, and the findings may not be generalizable. The decision to initiate V–V ECMO was influenced by institutional expertise and available resources. Although early ECMO may be beneficial in selected patients with drowning-associated cardiac arrest, the current eligibility criteria remain important, and further studies are needed to clarify the optimal indications and timing.

## Data Availability

The datasets used and/or analysed during the current study are available from the corresponding author on reasonable request.

## Abbreviations

ARDS: Acute respiratory distress syndrome
CT: Computed tomography
GCS: Glasgow Coma Scale
V–V: ECMO Venovenous extracorporeal membrane oxygenation
